# Identification of NADPH oxidase family members associated with cold stress in strawberry

**DOI:** 10.1002/2211-5463.12393

**Published:** 2018-02-20

**Authors:** Yunting Zhang, Yali Li, Yuwei He, Wenjie Hu, Yong Zhang, Xiaorong Wang, Haoru Tang

**Affiliations:** ^1^ College of Horticulture Sichuan Agricultural University Chengdu China; ^2^ Institute of Pomology and Olericulture Sichuan Agricultural University Chengdu China

**Keywords:** cold stress, expression profiles, NADPH oxidase, phylogenetic analysis, *Rbohs*, strawberry

## Abstract

NADPH oxidase is encoded by a small gene family (Respiratory burst oxidase homologs, *Rbohs*) and plays an important role in regulating various biological processes. However, little information about this gene family is currently available for strawberry. In this study, a total of seven *Rboh* genes were identified from strawberry through genomewide analysis. Gene structure analysis showed the number of exons ranged from 10 to 23, implying that this variation occurred in *FvRboh* genes by the insertion and distribution of introns; the order and approximate size of exons were relatively conserved. FvRbohC was predicted to localize to the thylakoid membrane of the chloroplast, while other members were computed to localize to the plasma membrane, indicating different functions. Amino acid sequence alignment, conserved domain, and motif analysis showed that all identified FvRbohs had typical features of plant Rbohs. Phylogenetic analysis of Rbohs from strawberry, grape, Arabidopsis, and rice suggested that the FvRbohs could be divided into five subgroups and showed a closer relationship with those from grape and Arabidopsis than those from rice. The expression patterns of *FvRboh* genes in root, stem, leaf, flower, and fruit revealed robust tissue specificity. The expression levels of *FvRbohA* and *FvRbohD* were quickly induced by cold stress, followed by an increase in NADPH oxidase activity, leading to $O_{2}^{-}$ accumulation and triggering the antioxidant reaction by the transient increases in SOD activity. This suggested these two genes may be involved in cold stress and defense responses in strawberry.

**Abbreviations**
APX: ascorbate peroxidase
CAT: catalase
CTAB: cetyltrimethylammonium bromide
EDTA: ethylenediaminetetraacetic acid
H_2_O_2_: hydrogen peroxide
MDA: malondialdehyde
$O_{2}^{-}$: superoxide anion
OH^.^: hydroxyl radical
POD: peroxidase
Rbohs: respiratory burst oxidase homologs
ROS: reactive oxygen species
SOD: superoxide dismutase

NADPH oxidase, an enzyme complex that catalyzes the NADPH‐dependent one‐electron reduction of molecular oxygen to the superoxide anion $O_{2}^{-}$, is also responsible for the accumulation of reactive oxygen species (ROS) associated with an abrupt rise in oxygen consumption as the respiratory burst oxidase 1, 2, 3. The oxidase complex comprises a membrane‐bound heterodimer, called flavocytochrome *b558*, consisting of gp91^phox^ and p22^phox^, and four cytosolic components (p47^phox^, p67^phox^, p40^phox^, and the small GTPase Rac) in phagocytes. Once the cell is stimulated, cytosolic components will interact with flavocytochrome *b558* to activate NADPH oxidase and then generate $O_{2}^{-}$, subsequently ending with the secondary production of other ROS such as hydrogen peroxide (H_2_O_2_) and hydroxyl radical (OH^.^) 4, 5, 6. ROS plays an important role in immunity and cell growth, but excessive accumulation of ROS can cause cellular damage and might be toxic 7, 8. Still, some evidences have shown that ROS at low concentration could be the ‘alarm’ signal to activate defense responses in plants 9, 10. A balance between generation and elimination of the free radicals can keep cellular ROS homeostasis. Plants have evolved complex antioxidant defense systems including nonenzymatic (low molecular weight antioxidant compounds) and enzymatic (SOD, CAT, POD, and APX) components that scavenge excessively accumulated ROS under stress conditions 11, 12.

In plants, NADPH oxidase is known as respiratory burst oxidase homologs (Rbohs), which encodes a homolog of mammalian phagocyte *gp91* ^*phox*^. It was proved that plant Rbohs are plasma membrane enzymes. The main structure of Rbohs contains six conserved transmembrane domains with C‐terminal FAD and NADPH hydrophilic domains, two heme groups, and two N‐terminal Ca^2+^‐binding EF‐hand motifs which account for being regulated by Ca^2+^ 1. *Rbohs*, a small gene family, have been identified and isolated in a wide range of plants. *Arabidopsis* has ten members (*AtRbohA–J*), with a tissue‐specific expression pattern: *AtRbohA–G* and *AtRbohI* in the roots, *AtRbohH* and *AtRbohJ* in pollens, and *AtRbohD* and *AtRbohF* throughout the plant, suggesting that differential expression profile and functions are formed in plant growth 1, 13, 14. NADPH oxidase has not only been linked with plant development, but also responds to different abiotic or biotic stress conditions mainly by adjusting the ROS generation 15. ROS signals derived by orthologs of two *AtRbohs* (A*tRbohH* and *AtRbohJ*) are involved in pollen tube growth in tobacco 16. Interestingly, the same function was not testified in *Arabidopsis thaliana*. Moreover, Rboh‐dependent ROS generation has been associated with root and hypocotyl elongation, stomatal movement, seed germination, and fruit ripening 17, 18. Several studies have evinced that NADPH oxidases mediate an oxidative burst to respond to stress. The tomato *SlRboh1* (homologous to *AtRbohF*) expression level increased, followed by much ROS accumulation, to adapt to the higher CO_2_ concentration environment 19, and it was also related to the regulation of stomatal movements in endurance to high temperature stress mediated by the phytohormones, abscisic acid (ABA), and brassinosteroid (BR) 20, 21. *AtRbohD* could affect H_2_O_2_ accumulation and involved in the release from suppression of root elongation by ethylene signaling during hypoxic stress 22. In most instances, an inhibition of NADPH oxidase contributes to the decrease in ROS production, then leading to the change of reaction to cell death and stress resistance. For example, DPI (diphenylene iodonium) inhibited NADPH oxidase activity, which decreased the production of H_2_O_2_ so as to lower ethylene‐induced cell death rates in rice 23 and resulted in diminishment of expression of several defense‐related genes in response to abiotic stress, such as wounding, oligosaccharides, systemin, and methyl jasmonate in *Lycopersicon esculentum* 24.

Strawberry is a perennial herb belonging to berry fruit crops. It is a model plant with high economic and nutritional value in *Rosaceae* genomics research. However, strawberry is often subjected to extreme sporadic chilling injury in the short term, which leads to huge economic loss because insulation measures are not strictly applied. Based on the significance of NADPH oxidase in the regulation of plant growth, development, and adaption to the environment, in this study, we identified NADPH oxidase family members from strawberry and analyzed their conserved motif, homology, and phylogenetic relationship with other plants. Enzyme activity and expression profiles in response to cold stress are also presented to provide scientific and theoretical basis for strawberry cultivation.

## Materials and methods

### Plant materials and treatments

Different tissues (root, stem, leaf, flower, and ripe fruit) of strawberry (*Fragaria×ananassa* cv. Toyonaka) were collected to verify NADPH oxidase family genes' tissue‐specific expression. Meanwhile, the strawberry seedlings from current‐year stem tip were grown in 12 cm × 10 cm pots filled with a 1 : 1 (v/v) mixture of soil and perlite, subjected to two‐month routine management from March 2014 in the greenhouse of Sichuan Agricultural University. Subsequently, the potted seedlings with vigorous and uniform growth status were used in the following experiment. They were moved to growth chambers (RXZ‐260B) with controlled environmental conditions (25 ± 1 °C, 108 μmol·m^−2^·s^−1^·16 h·d^−1^, and 70 ± 5% relative humidity) for 2 weeks. After that, the plants were subjected to cold stress at 4 °C. The samples (leaves) were collected at 0, 6, 12, 24, 48, 72, and 96 h after cold stress treatment and prepared in triplicate.

### Identification and annotation of FvRboh homologs in the strawberry genome

Ten protein sequences of Arabidopsis AtRbohA–J obtained from The Arabidopsis Information Resource (https://www.arabidopsis.org/) and simultaneously the Hidden Markov Model (HMM) profile of NADPH_Ox (PF08414) downloaded from the Pfam protein family database (http://pfam.sanger.ac.uk) were used as queries to search against the strawberry genome v.1.0 hybrid gene proteins. Subsequently, protein, gene, and cDNA sequences were all retrieved and examined using the National Center for Biotechnology Information (NCBI) BLAST tool with the default cutoff parameters.

### Bioinformatics analysis

The exon–intron structure of the *FvRbohs* was identified using the online Gene Structure Display Server v.2.0 (http://gsds.cbi.pku.edu.cn/) based on alignments of their coding sequences with corresponding genomic sequences. The protein molecular weight, theoretical pI, instability, aliphatic index, and grand average of hydropathicity (GRAVY) index were obtained using ExPASy ProtParam tool (http://web.expasy.org/protparam/). Putative signal peptides and subcellular location were predicted in the SignalP 4.1 Server (http://www.cbs.dtu.dk/services/SignalP/) and ProtComp v.9.0 (http://linux1.softberry.com/berry.phtml?topic=protcomppl&group=programs&subgroup=proloc). DNAMAN v.8.0 software was used to perform multiple sequence alignment of FvRboh protein amino acid sequences. Additionally, SMART program (http://smart.embl-heidelberg.de), NCBI CDD (https://www.ncbi.nlm.nih.gov/Structure/cdd/cdd.shtml), TMHMM Server v.2.0 (http://www.cbs.dtu.dk/services/TMHMM/), and TMpred (http://www.ch.embnet.org/software/TMPRED_form.html) were applied to speculate EF‐hands, transmembrane domains (TMs), and conserved binding sites for flavin adenine dinucleotide (FAD), NAD pyrophosphate, and NADP ribose. Phylogenetic relationship of Rbohs between strawberry and other species was constructed using Clustal X v.2.0 and MEGA v.6.0 software with the neighbor‐joining (NJ) method under the Poisson model, and 1000 bootstrap test replicates would evaluate the reliability of interior branches. The basic sequence information for bioinformatics analysis was described in Appendix S1.

### NADPH oxidase extraction and assays

A two‐phase aqueous polymer partition system was used to isolate the leaf plasma membranes. The purity of the plasma membrane was assessed by assaying the activities of marker enzymes (vanadate‐sensitive ATPase). A total of one milliliter reaction mixture contained 50 mm Tris/HCl buffer (pH 7.5), 0.5 mm XTT, 100 μm EDTA, 15–20 μg membrane protein, and 100 μm NADPH. After the addition of 100 μm NADPH, the reaction was monitored at 470 nm with absorbance coefficient of 21.6 mm ^−1^·cm^−1^. The NADPH oxidase activity was assayed based on the reduction of XTT by $O_{2}^{-}$ radicals 25, 26. Corrections were made for background production in the presence of 50 units of SOD.

### Expression profiles of *FvRbohs*

Total RNA was extracted from samples using the improved CTAB method 27. After quality assessment, 1 μg of total RNA was reverse‐transcribed into the complementary DNA (cDNA) with PrimeScript™ RT reagent Kit with gDNA Eraser (Perfect Real Time) (Takara, Japan) according to the manufacturer's instructions. The expression level of NADPH oxidase genes in different tissues and cold stress was determined by semiquantitative RT‐PCR and quantitative RT‐PCR, respectively. All quantitative real‐time PCRs were performed using SYBR Green Premix Ex Taq™ (Takara, Japan) on the CFX96 real‐time PCR system (Bio‐Rad, USA) in triplicate of each sample. Total 20 μL reaction contained 0.6 μL of each primer (10 μm), 10 μL SYBR Premix (Takara, Japan), 2 μL cDNA (10 ng) template, and 6.8 μL of RNase‐free water. Reaction protocol was set with two‐step cycling conditions: 95 °C for 3 min, followed by 40 cycles of 95 °C for 10 s, and 60 °C for 30 s. Fluorescence was monitored at the end of the annealing step each cycle. Melting curve was inserted, ramping from 65 °C to 95 °C (increment 0.5 °C/5 s) after the final cycle. The relative expression level was analyzed with the 2^−▵▵Ct^ method. *FaActin* was used as the reference gene to standardize the raw data. Primers for *Rbohs* and *Actin* genes are listed in Table 1.

**Table 1:** Primers for semiquantitative RT‐PCR and quantitative RT‐PCR

Gene name	Forward primers (5′ to 3′)	Reverse primers (5′ to 3′)	Product size (bp)
*FvRbohA*	CTCGTCCAATAGTAGAATCC	ATTATTCTGAGAAGCAATCG	172
*FvRbohB*	GGATATGAGACAGTGAAGAT	GAAGTAATTGAGAACGGATG	164
*FvRbohC*	CCAGAAGATCATATCGGAGAAG	CTCGTTGTCGGAGTGATACT	75
*FvRbohD*	TGTTGATGACCATAGCATTC	AGGAGAGCGTAGACTATAAC	142
*FvRbohE*	TATAATGCTGAGTGCTTCTG	TGGTCTGCTATAGTCTATGTAA	172
*FvRbohF*	TGGCGACGAGCATGGATAGTTT	AGGGTTTCAGCAGCACCTTTGG	143
*FvRbohH*	TGCACGGTCTGCGCTTATTA	TCCGGCTTTGTGAGACAACA	80
*FvActin*	TTCACGAGACCACCTATAACTC	GCTCATCCTATCAGCGATT	122

### Determination of $O_{2}^{-}$ production rate and SOD enzyme activity

A total of 0.1 g of the leaf powder was homogenized in 1 mL ice‐cold potassium phosphate buffer (50 mm, pH 7.8) containing 1% (w/v) of polyvinylpolypyrrolidone (PVP). The homogenate was centrifuged at 10 000 × ***g*** for 10 min at 4 °C. The supernatant fraction was prepared for determination of $O_{2}^{-}$ content and SOD activity. Total soluble protein contents of the extracts were determined according to Bradford 28, and bovine serum albumin was used as a standard.

Superoxide anion ($O_{2}^{-}$) was determined according to the method of Cai *et al*. 29. The supernatant (0.5 mL) was mixed with 0.5 mL of 50 mm potassium phosphate buffer (pH 7.8) and 1 mL of 1 mm hydroxylamine hydrochloride and then incubated at 25 °C for 1 h. After incubation, 1 mL of 17 mm *p*‐aminophenylsulfonic acid (in glacial acetic acid/H_2_O (3 : 1)) and 1 mL of 7 mm α‐naphthylamine (in glacial acetic acid/H_2_O (3 : 1)) were added into the mixture for a further 20 min at 25 °C, followed by immediate measurement of absorbance at 530 nm. A standard curve with ${\mathrm{NO}}_{2}^{-}$ was used to calculate the production rate of $O_{2}^{-}$ from the chemical reaction of $O_{2}^{-}$ and hydroxylamine hydrochloride.

Total SOD (http://www.chem.qmul.ac.uk/iubmb/enzyme/EC1/15/1/1.html) activity was measured by monitoring the inhibition of photochemical reduction of nitro blue tetrazolium chloride (NBT) 30, 31. The 3 mL of reaction mixture was comprised of 50 mm potassium phosphate buffer (pH 7.8), 13 mm methionine, 75 μm NBT, 2 μm riboflavin, 100 μm EDTA, and 100 μL of enzyme extract. Subsequently, the reaction mixtures were illuminated under light intensity of 80 μmol·m^−2^·s^−1^ at 25 °C for 15 min. One unit of SOD activity was defined as the amount of enzyme that was required to cause 50% inhibition of the reduction of NBT as monitored at 560 nm.

### Lipid peroxidation

Lipid peroxidation was estimated by determining the malondialdehyde (MDA) contents in the leaves using the thiobarbituric acid method 32, 33. A total of 0.2 g of leaf samples was homogenized in 4 mL of 10% (w/v) trichloroacetic acid (TCA). The homogenate was centrifuged at 10 000 × ***g*** for 10 min. An aliquot of 2 mL supernatant was mixed with 2 mL of 0.5% (w/v) 2‐thiobarbituric acid (TBA) made in 10% TCA. The mixture was boiled at 100 °C for 10 min and then quickly cooled on ice. Samples were centrifuged at 10 000 × ***g*** for 10 min. The supernatant absorbance was monitored at 450, 532, and 600 nm, respectively, and MDA concentration was expressed as μmol·g^−1^ fresh weight.

## Results

### Identification and gene structure of *Rboh* homologs in the strawberry genome

A total of seven Rboh genes were identified and then named *FvRbohA*,* FvRbohB*,* FvRbohC*,* FvRbohD*,* FvRbohE*,* FvRbohF*, and *FvRbohH* according to conserved domain and multiple sequence alignment with *Arabidopsis* (Table 2). As shown, five genes except for *FvRbohC* and *FvRbohH* were all mapped to a specific chromosome (1, 5, and 6, respectively). The length of 9381‐bp *FvRbohA* and 12025‐bp *FvRbohC* genes was much longer than that of other members. In addition, the open reading frame length ranged from 2376 to 5598 bp and deduced protein sequence lengths varied from 791 to 1865 amino acids.

**Table 2:** List of *FvRbohs* identified in strawberry

Gen name	Gene ID	Chromosome	Location	Gene length (bp)	ORF length (bp)	Amino acid length (aa)
*FvRbohA*	gene31855	chr5	1932913–1942293	9381	3084	1027
*FvRbohB*	gene22214	chr6	5063705–5067964	4260	2661	886
*FvRbohC*	gene01814	–a	–a	12025	5598	1865
*FvRbohD*	gene00215	chr5	6658520–6662290	3771	2808	935
*FvRbohE*	gene12928	chr1	7411220–7415355	4136	2649	882
*FvRbohF*	gene26084	chr5	7980576–7984305	3730	2376	791
*FvRbohH*	gene14024	–a	–a	4296	2598	865

a Unplaced scaffold.

**Figure 1:**
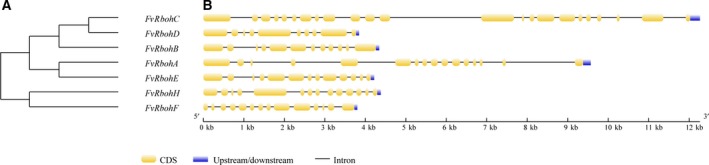
Phylogenetic tree (A) and exon–intron structures (B) of *FvRboh* genes. A phylogenetic tree of *FvRboh* genes was constructed with the neighbor‐joining method with 1000 bootstrap test replicates. Exons, introns, and untranslated regions (UTRs) are indicated by yellow boxes, gray horizontal lines, and blue boxes, respectively. The scale bar represents 12 kb.

The unrooted phylogenetic tree showed that *FvRboh* genes were placed in two well‐resolved clades. Five members including *FvRbohA*,* FvRbohB*,* FvRbohC*,* FvRbohD,* and *FvRbohE* shared one clade, which indicated they might share a common evolutionary history. The other clade only contained *FvRbohH* and *FvRbohF* (Fig. 1A). The number of exons ranged from 10 in *FvRbohD* to 23 in *FvRbohC*. *FvRbohF* and *FvRbohH* owned 14 exons, while other genes had different number of exons. Although the order and approximate size of exons among the *FvRbohs* were relatively conserved, the length of introns was variable, which lead to a diversity of gene structures. In particular, *FvRbohC* and *FvRbohA*, respectively, contained one and four long introns consistent with their big size (Fig. 1B).

### Amino acid sequence and conserved motif analysis of FvRbohs

The relative molecular mass of the seven predicted FvRboh proteins ranged from 90.20 KDa (FvRbohF) to 206.97 KDa (FvRbohF), however, with similar corresponding isoelectric point around 9.0. Instability indexes higher than 40 showed that all family members of FvRbohs were unstable protein. Aliphatic indexes were predicted between 85.84 and 92.10. FvRbohs are inclined to be hydrophilic because grand average of hydropathy (GRAVY) negative values varied from −0.117 to −0.275. These proteins had no signal peptides, but contained six transmembrane helices (TMHs). Subcellular localization results indicated that most of the FvRbohs were localized to the plasma membrane, while only FvRbohC was computed to reside in the chloroplast thylakoid membrane (Table 3).

**Table 3:** Protein properties of FvRbohs

Protein	MW (kDa)	pI	Instability index	Aliphatic index	GRAVY	SignalP	TMHs	Location
FvRbohA	116.25	8.94	50.16	86.83	−0.255	No	6	Plasma membrane
FvRbohB	101.02	8.93	40.47	92.10	−0.170	No	6	Plasma membrane
FvRbohC	206.97	8.88	46.45	88.53	−0.240	No	6	Chloroplast thylakoid membrane
FvRbohD	105.46	9.14	40.47	85.84	−0.275	No	6	Plasma membrane
FvRbohE	100.03	8.63	48.85	87.61	−0.174	No	6	Plasma membrane
FvRbohF	90.20	8.74	50.13	85.94	−0.117	No	6	Plasma membrane
FvRbohH	98.37	8.93	43.79	88.81	−0.159	No	6	Plasma membrane

**Figure 2:**
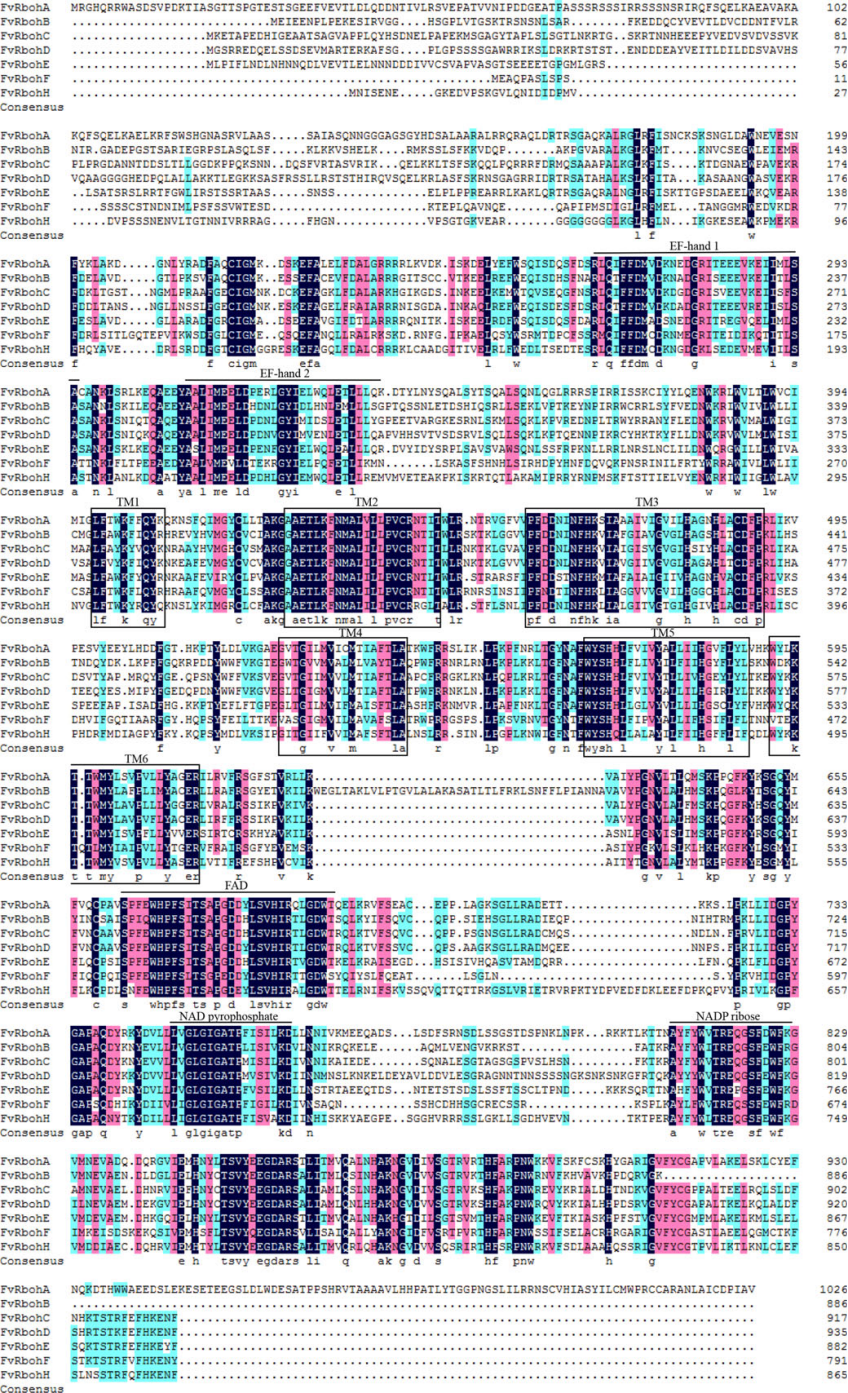
Protein sequence multi‐alignment and domain structure of the Rbohs from strawberry. Conservative residues are highlighted by black shadings, and a lower level of conservations is indicated by lighter shadings. EF‐hands and conserved binding sites for FAD, NAD pyrophosphate, and NADP ribose are represented by straight lines. Transmembrane domains are indicated by boxes.

**Figure 3:**
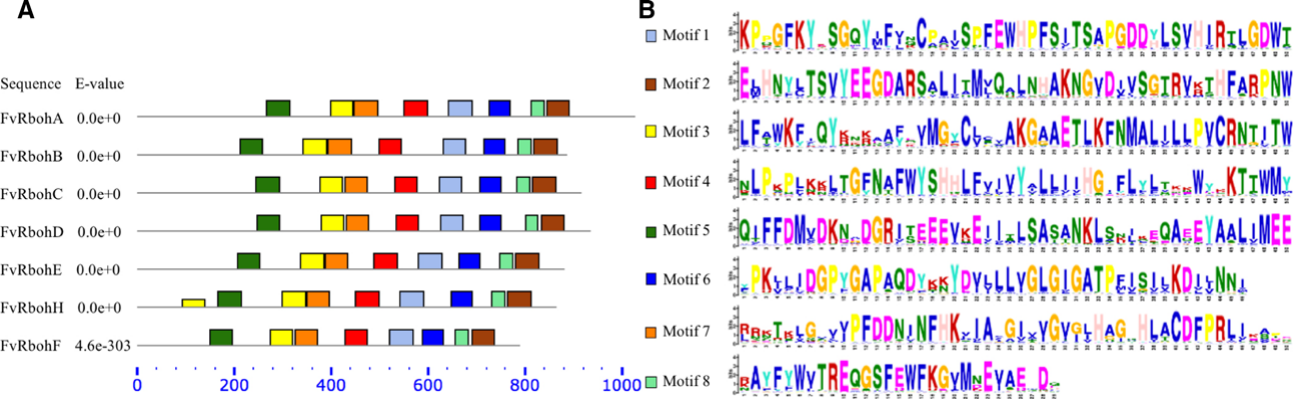
Conserved motifs distribution (A) and sequence (B) in FvRboh proteins. Motif of the FvRboh family members in strawberry was analyzed using the MEME web server. Different color boxes represent eight putative motifs, respectively. Names of all family members and *E*‐values are shown on the left side. The blue dotted line scale represents the length of amino acid. The height of a letter indicates its relative frequency at the given position in the motif.

In addition, multiple sequence alignment of the seven FvRboh proteins was performed (Fig. 2), which demonstrated that these sequences were highly conservative and contained typical conserved domains of NADPH oxidase including two putative Ca^2+^‐binding EF‐hands and six transmembrane domains (TM1–6) in the N‐terminal region, and flavin adenine dinucleotide (FAD), NAD pyrophosphate, and NADP ribose conserved binding sites in the C‐terminal region. To further reveal the structural diversity and function prediction of FvRboh proteins, a total of eight conserved motifs were identified using online MEME tool and annotated based on Pfam, NCBI CDD, and PROSITE databases (Fig. 3). All seven FvRbohs shared eight motifs once, except for motif 3 in FvRbohH. Moreover, the order and distribution of the eight motifs in these proteins were almost the same as described above (Fig. 3A). Motif 1 was annotated as FAD‐binding domain; motifs 2, 6, and 8 were annotated as NAD‐binding region; and motif 5 was annotated as EF‐hands (Fig. 3B).

### Phylogenetic analysis of Rbohs in different plants

**Figure 4:**
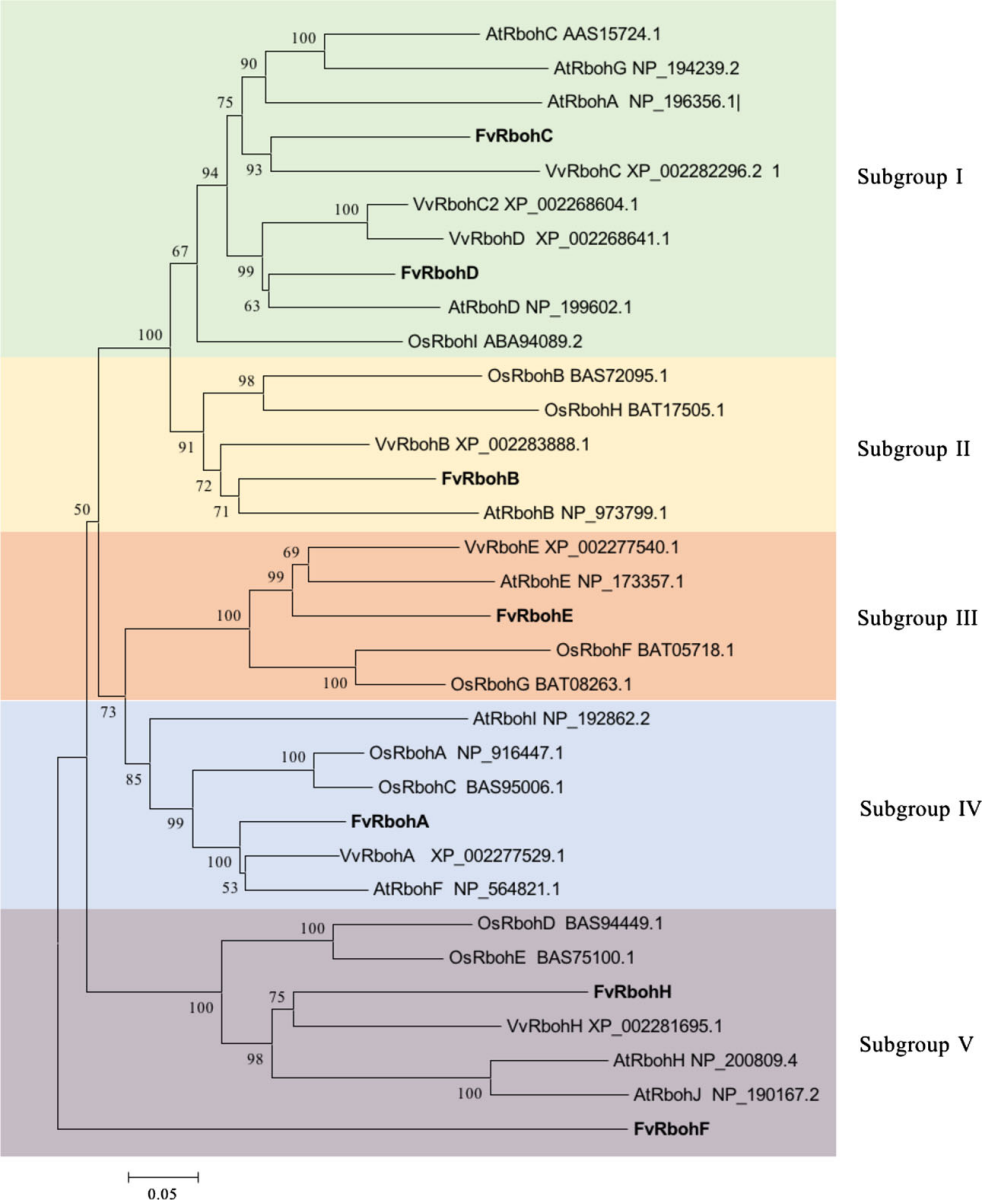
Phylogenetic analysis of Rbohs in strawberry, grape, Arabidopsis, and rice. The phylogenetic tree was constructed with the neighbor‐joining method based on the Poisson model. Tree reliability was assessed using 1000 bootstrap replicates. The numbers indicated for each clade represent bootstrap support values given as percentages. Five subgroups are shown as I, II, III, IV, and V.

To analyze phylogenetic relationship of NADPH oxidase family members in strawberry, grape, Arabidopsis, and rice, the amino acid sequences of 33 Rboh proteins, including 7 from strawberry, 7 from grape, 10 from Arabidopsis, and 9 from rice, were aligned and used to construct an unrooted phylogenetic tree with the neighbor‐joining method (Fig. 4). The results indicated that 33 Rbohs could be assigned to five distinct subgroups (I, II, III, IV, and V). FvRbohC and FvRbohD were divided into the subgroup I. FvRbohF and FvRbohH were classified into the subgroup V. FvRbohA, FvRbohB, and FvRbohE belonged to subgroups IV, II, and III, respectively. Notably, FvRbohs were clustered together with grape or Arabidopsis Rbohs first, suggesting that strawberry Rboh proteins were more closely related to those from grape and Arabidopsis than to those of rice, which was consistent with the fact that strawberry, grape, and Arabidopsis are eudicots and diverged more recently from a common ancestor than from the lineage leading to monocots.

### NADPH oxidase activity and expression patterns of *FvRbohs* in response to cold stress

**Figure 5:**
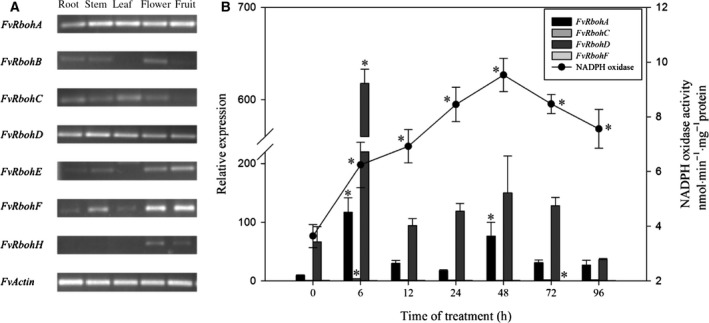
Expression patterns of *FvRbohs* in different tissues (A) and response to cold stress (B). The left and right *Y*‐axis is the scale of the relative transcript abundance level and NADPH oxidase activity, respectively. The *X*‐axis is the time course of 4 °C cold treatment. Values represent means ± standard error from three biological experiments (*n* = 3). Asterisks indicate significant differences based on one‐way ANOVA in SPSS 23.0 followed by the Dunnett *t*‐test (time 0 as the control, *P* < 0.05).

Expression of *Rbohs* in plant as previously reported was tissue‐specific. In our study, the results indicated *FvRbohA*,* FvRbohC*,* FvRbohD*, and *FvRbohF* could be detected in root, stem, leaf, flower, and fruit. *FvRbohB* and *FvRbohE* were expressed in root, stem, flower, and fruit, while *FvRbohH* was only observed in flower and fruit (Fig. 5A). Exposure to cold stress, NADPH oxidase activity in strawberry leaves showed a tendency: increased quickly and peaked at 48 h, and then had a decrease. Although the enzyme activity decreased in the late phase of treatment, it was still higher than that of the initial phase (25 °C). The relative expression levels of four members (*FvRbohA, FvRbohC, FvRbohD,* and *FvRbohF*) that could be detected in strawberry leaves were examined to explore their response to cold stress by real‐time qPCR analysis. As shown, *FvRbohA* and *FvRbohD* were keeping high expression levels, while *FvRbohC* and *FvRbohF* were difficult to be tested during cold treatment. Generally, the expression patterns of *FvRbohA* and *FvRbohD* were observed to be M‐shaped. The transcript abundances of *RbohA* and *RbohD* were highly induced and reached the maximum value after the first 6‐h low‐temperature exposure (Fig. 5B), so the peak value showed up earlier than that of NADPH oxidase activity.

### $O_{2}^{-}$ production rate, SOD enzyme activity, and lipid peroxidation

**Figure 6:**
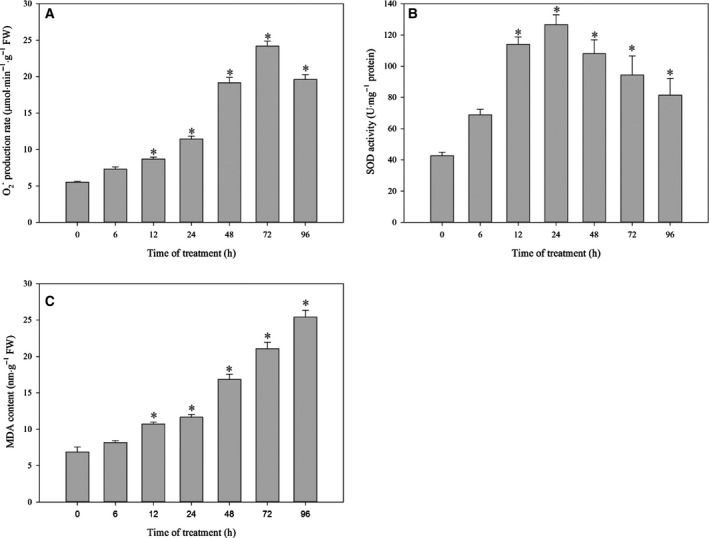
$O_{2}^{-}$ production rate (A), SOD enzyme activity (B), and lipid peroxidation (C). Each value represents mean ± standard error in histogram. Values represent means ± standard error from three biological experiments (*n* = 3). Asterisks indicate significant differences based on one‐way ANOVA in SPSS 23.0 followed by the Dunnett *t*‐test (time 0 as the control, *P* < 0.05).

$O_{2}^{-}$ production rate increased slowly at the initial stage of cold stress and then followed a transient burst. After 72 h, a slight decrease in $O_{2}^{-}$ production occurred (Fig. 6A). The change trend of $O_{2}^{-}$ production rate was almost coinciding with NADPH oxidase activity. SOD activity had a quick increase and reached the peak value at 24 h, which inhibited the $O_{2}^{-}$ production. However, at the late stage of cold stress, SOD activity began to decrease (Fig. 6B). The MDA accumulation was often used as an indicator of lipid peroxidation. In general, the successive cold stress for 96 h caused a linear increase in MDA content (Fig. 6C).

## Discussion

It was well known that ROS was toxic to biological organisms by oxidizing lipids, proteins, DNA, and carbohydrates, resulting in breakdown of normal cellular, membrane, and reproductive functions 34, but ROS when its concentration is in the appropriate range can act as a signal molecule to influence cell growth, organ development, and defense responses. In plant, NADPH oxidase‐catalyzed conversion of the superoxide anion ($O_{2}^{-}$) to other ROS, such as hydrogen peroxide, hydroxyl radicals, and perhydroxyl radicals, was the major source of ROS production 26. NADPH oxidase was coded by a small gene family of *Rbohs*. Benefiting from the availability of whole‐genome sequence in recent years, so far, a few model and crop plant NADPH oxidase families have been identified at the genomewide level, including 10 in *Arabidopsis* 1, 9 in rice 35, 6 in barley 36, 9 in apple 37, and 7 in grape 38. However, very little is known about this family in strawberry.

This study comprehensively identified and characterized seven *FvRboh* genes from strawberry at the genomewide level, which are mainly distributed in chromosomes 1, 5, and 6. Gene structure analysis showed *FvRbohs* included at least ten exons; *FvRbohC* had the most exons of 23, which was greatly surpassed that in other plants. Actually, the majority of *Rbohs* harbored 10–14 exons in *Arabidopsis*, rice, barley, and grape, except that *AtRbohD* contained eight exons and *OsRbohD*,* VvRbohB*, and *VvRbohD* had 15 exons in their coding DNA sequence (CDS) 39. The large structural variation of *Rboh* genes implied the significant genomic change during their evolutionary history, possibly as a result of highly diverse distribution and insertion of intronic regions amid the exonic sequences. In addition, FvRbohC protein was predicated to localize to the thylakoid membrane of the chloroplast, while other members were computed to localize to the plasma membrane, indicating the different functions. This case was also reported in the grape 38, in contrast to *Arabidopsis* and rice, where all the Rbohs were predicted to localize to the plasma membrane. The alignment of seven FvRbohs clearly showed the presence of two EF‐hands, six TMs, FAD, and NAD‐binding sites, which were known to be present in Rbohs from other plant species. EF‐hand regions that were absent from the mammalian phagocyte gp91^phox^ protein can bind Ca^2+^, accounting for the direct regulation of plant Rbohs by Ca^2+^ 1, 14, 40. Thus, the regulation way of NADPH oxidases in plant might be different from that in mammalian phagocytes. Subsequently, the characterization and distribution of conserved motifs furtherly emphasized the importance of these structures. The phylogenetic tree of Rboh proteins from different plant species showed FvRbohs had a closer correlation with VvRbohs and AtRbohs than those from monocotyledonous rice, indicating the genes were established prior to the divergence of the corresponding taxonomic lineages. Moreover, those homologs clustered in the same group were possible to be involved in similar functions; however, further experimental analyses are necessary to confirm this.

The tissue‐specific expression patterns of *Rboh* genes have been reported in some species; however, there is no uniform expression pattern for the plant Rboh genes reported. In grape, seven *VvRboh* genes could be detected in all tissues involved in young leaves, roots, stems, inflorescences, berries, tendrils, and ovules 38, while four of the six and two of the ten Rboh genes had widespread constitutive spatial expression patterns in barley and Arabidopsis, respectively 1, 36. In our study, *FvRbohA*,* FvRbohC*,* FvRbohD*, and *FvRbohF* were tested in all tissues; *FvRbohA* and *FvRbohD* were the highly expressed genes. *FvRbohB* and *FvRbohE* had transcript abundances in most of the tissues except for leaves, while *FvRbohH* was expressed only in flower and fruit. These findings might suggest tissue‐specific function of these family members.

It has been documented that low temperature, as many other stressful environmental conditions, could trigger enhanced generation of ROS to disrupt cellular homeostasis. But ROS when its concentration is in the appropriate range can act as a signal molecule to initiate defense responses 41. Plasma membrane, peroxisomes, chloroplasts, and mitochondria are potential sources of ROS in plant cells 42. The plasma membrane‐located NADPH oxidases have been shown to mainly involve in ROS production and play critical roles in plant development and defense responses 15, 43. Our study showed that NADPH oxidase activity increased sharply at the early stage of strawberry seedlings exposed to cold stress and then had a slight decline, but still kept high level, compared to 0 h (25 °C), accompanied by the production of the superoxide anion. To alleviate the negative effects of stress and balance the ROS level, organism would initiate enzymatic and nonenzymatic mechanisms to maintain the cellular redox homeostasis. SOD was involved in dismutation of $O_{2}^{-}$ to H_2_O_2_, and its activity reflected the ability to adapt to stress in plant. In our study, SOD activity increased at the initial treatment and then decreased, which demonstrated that short‐term cold stress could induce antioxidative defense mechanism in the plant. However, the longer the stress time was, the more ROS produced, so that antioxidative enzyme activity was impaired and the ROS could not be removed effectively. Subsequently, peroxidation of lipids in the cell membrane resulted in a massive MDA accumulation, which was toxic to plant 44. Correspondingly, the *Rboh* genes (*FvRbohA*,* FvRbohC*,* FvRbohD*, and *FvRbohF*) which were specifically expressed in leaves were monitored during the cold treatment. Fv*RbohA* and *FvRbohD* reacted quickly to cold stress by improving transcript levels, while *FvRbohC* and *FvRbohF* were keeping a low expression level during this process. *CsRbohA* was upregulated by low temperature in short time, which was consistent with our findings. *AtRbohD* in *Arabidopsis thaliana* was constitutively and ubiquitously expressed and showed a high degree of stress responsiveness 45, 46. Recently, it was reported that the expression level of *AtRbohD* had an extremely significant increase at an early stage in the hypoxia response. *AtRbohD*‐knockout mutant was used to further demonstrate that *AtRbohD* played a key role in regulating the transcript abundance of downstream hypoxia‐inducible genes at an early stage during hypoxic stress and could be a cross talk in ethylene modulating H_2_O_2_ signal transduction in the hypoxia response pathway 22. These facts hinted that *FvRbohA* and *FvRbohD* might be the dominate induction of antioxidant defense system in response to cold stress.

## Conclusion

In this study, we totally identified seven Rboh genes from the strawberry genome and revealed classification, gene structure, evolution, conserved protein motif, and phylogenetic relationship by systematical bioinformatic analysis. NADPH oxidase was related to initiation of antioxidant system in leaves of strawberry against cold stress by regulating $O_{2}^{-}$ production. Expression profile analysis among different tissues (root, stem, leaf, flower, and fruit) showed that *FvRboh* genes had the tissue‐specific characteristic. Furthermore, *FvRbohA* and *FvRbohD* maintained the high expression in response to cold stress, implying that they played a crucial role in this process. This information provides some insights into potential functions of strawberry *Rbohs* and increases our understanding of the molecular basis of the acquired cold tolerance of strawberry and even the adaptability of strawberry to other stress conditions.

## Author contributions

YTZ and HRT conceived and designed the experiment. YTZ, YLL, and YWH performed the experiment and analyzed the data. WJH collected the samples. YTZ wrote the manuscript. YZ, XRW, and HRT reviewed drafts of the manuscript. All authors read and approved the final version.

## Supporting information


**Appendix S1.** Basic sequence information for bioinformatics analysis.
